# Targeted Gold Nanoparticle–Oligonucleotide Contrast Agents in Combination with a New Local Voxel-Wise MRI Analysis Algorithm for In Vitro Imaging of Triple-Negative Breast Cancer

**DOI:** 10.3390/nano9050709

**Published:** 2019-05-07

**Authors:** Rajat Chauhan, Nagwa El-Baz, Robert S. Keynton, Kurtis T. James, Danial A. Malik, Mingming Zhu, Ayman El-Baz, Chin K. Ng, Paula J. Bates, Mohammad Tariq Malik, Martin G. O’Toole

**Affiliations:** 1 Department of Bioengineering, University of Louisville, Louisville, KY 40292, USA; rajat.rajat@louisville.edu (R.C.); Kurtis.james@louisville.edu (K.T.J.); ayman.elbaz@louisville.edu (A.E.-B.); 2 Department of Pharmacology and Toxicology, University of Louisville, Louisville, KY 40202, USA; nagwa.el-baz@louisville.edu (N.E.-B.); danial.malik@louisville.edu (D.A.M.); 3 Department of Radiology, University of Louisville, Louisville, KY 40202, USA; mingming.zhu@louisville.edu (M.Z.); chin.ng@louisville.edu (C.K.N.); 4 Department of Medicine and James Graham Brown Cancer Center, University of Louisville, Louisville, KY 40202, USA; paula.bates@louisville.edu (P.J.B.); tariq.malik@louisville.edu (M.T.M.)

**Keywords:** AS1411, gadolinium based contrast agent, G-quadruplex, gold nanoparticles, MRI local voxel-wise analysis

## Abstract

Gold nanoparticles (GNPs) have tremendous potential as cancer-targeted contrast agents for diagnostic imaging. The ability to modify the particle surface with both disease-targeting molecules (such as the cancer-specific aptamer AS1411) and contrast agents (such as the gadolinium chelate Gd(III)-DO3A-SH) enables tailoring the particles for specific cancer-imaging and diagnosis. While the amount of image contrast generated by nanoparticle contrast agents is often low, it can be augmented with the assistance of computer image analysis algorithms. In this work, the ability of cancer-targeted gold nanoparticle–oligonucleotide conjugates to distinguish between malignant (MDA-MB-231) and healthy cells (MCF-10A) is tested using a T1-weighted image analysis algorithm based on three-dimensional, deformable model-based segmentation to extract the Volume of Interest (VOI). The gold nanoparticle/algorithm tandem was tested using contrast agent GNP-Gd(III)-DO3A-SH-AS1411) and nontargeted c-rich oligonucleotide (CRO) analogs and control (CTR) counterparts (GNP-Gd(III)-DO3A-SH-CRO/CTR) via in vitro studies. Remarkably, the cancer cells were notably distinguished from the nonmalignant cells, especially at nanomolar contrast agent concentrations. The T1-weighted image analysis algorithm provided similar results to the industry standard Varian software interface (VNMRJ) analysis of T1 maps at micromolar contrast agent concentrations, in which the VNMRJ produced a 19.5% better MRI contrast enhancement. However, our algorithm provided more sensitive and consistent results at nanomolar contrast agent concentrations, where our algorithm produced ~500% better MRI contrast enhancement.

## 1. Introduction

Nanoparticle systems are becoming increasingly popular for development as contrast agents for biomedical imaging due to the potential to combine multimodal imaging contrast, disease targeting, longer circulation times (compared to conventional small molecule agents), and engineered clearance pathways into a single entity. Among the many nanomaterials being developed, gold and gold-coated nanoparticles have attracted much attention due to their low toxicity, versatile surface chemistry, and tunable size. Furthermore, gold nanoparticles can display strong optical absorbance values due to the interaction between the electric field of light and the electron clouds of nanosized metallic objects (a phenomenon known as localized surface plasmon resonance or LSPR). Small GNPs, with diameters of ~4 nm have been shown to have enhanced permeability and retention (EPR) in tumor tissue making them ideal as therapeutic devices [1,2]. GNP can be conjugated with distinctive therapeutic and/or contrast agents allowing for a multimodal image guided drug delivery system [3,4,5,6]. DNA functionalization on GNPs has been shown to have efficient penetration into cancerous cells that further engenders contrast enhancement via their intracellular accumulation and decreased magnitude of longitudinal or transverse relaxation time [5,7]. Small GNPs (~5 nm) functionalized with guanine rich DNA oligonucleotide AS1411 (ACT-GRO-777) selectively constrains the growth of MCF-7 and MDA-MB-231 triple-negative Her2 receptor breast cancer cells without affecting nonmalignant MCF-10A breast epithelial cells [8,9]. Mechanistically, the AS1411 (ACT-GRO-777) forms a G-quadruplex structure that selectively binds nucleolin proteins present on the cell surface and in the cytoplasm of most types of cancer [10]. In healthy cells, nucleolin is produced, but localized in the nucleus. These non-nuclear nucleolin proteins found in cancer cells facilitate the survival of cancer cells via increasing the concentration of antiapoptotic mRNA and miRNA in the cytoplasm [11,12]. Moreover, the AS1411 functionalized spherical GNPs have higher cellular internalization and increased cytotoxicity in comparison to AS1411 alone, making them excellent candidates as a core structure for a cancer-targeted contrast agent [8,9].

Gadolinium(III) based contrast agents (GBCA) are extensively used in clinical magnetic resonance imaging (MRI) of soft tissues and tumor vasculature [13,14]. Currently, clinically used single-molecule-sized GBCAs are Gadoterate meglumine, Gadobutrol, Gadoteridol, Gadopentetate dimeglumine, Gadoversetamide, Gadodiamide, Gadofosveset trisodium, Gadoxetic acid, and Gadobenate dimeglumine (MultiHance) [15,16,17]. Unfortunately, there are some disadvantages to single-molecule GBCAs [18], including their relatively poor differentiation between benign and malignant tumors compared to their macromolecular conjugate constructs that show excellent tumor vascular hyperpermeability [19,20,21]. Analogous to the blood pool agents [18], the noncovalent interaction between a Gd^3+^-Ligand and its surrounding macromolecular construct increases its overall size, prevents its diffusion through vascular endothelium, and provides a longer imaging window due to reduced renal clearance, post intravascular injection. Efforts at improving the utility of GBCA’s typically focus on slowing Gd^3+^ rotational tumbling rate (*1/τ_r_*) and increasing Gd^3+^ inner sphere chemical exchange (*q*) with water molecules [22,23]. A series of investigations have been reported using GBCA chelation to macromolecular agents such as lipids [24], proteins [25], nucleic acids (DNA or RNA) [8,9,10,26], dendrimers [27], liposomes [28], aquasomes [29], and nanoparticles [2,4,30,31,32,33,34,35] for optimizing T1/T1-Weighted contrast as well as tumor targeting. These biodegradable macromolecules shorten the longitudinal (T1) relaxation of GBCAs, improve pharmacokinetics/pharmacodynamics, increase permeation through pores in tumor vasculature endothelium and reduce overall gadolinium renal toxicity via sterically shielding the toxic gadolinium center [2,4,8,9,10,24,25,27,28,29,30,31,32,33,34,35]. As a result, this study will also investigate chelation of GBCA’s to biodegradable macromolecules for targeted delivery due to their excellent tumor vascular hyperpermeability [19,20,21], pharmacokinetics/pharmacodynamics [8,9,10,25,26], conformational dynamics, and biodistribution [2,4,8,9,10,24,25,27,28,29,30,31,32,33,34,35]. In particular, our group has been working with nucleic acid-based macromolecules (DNA/RNA) such as biomolecule-targeting aptamers, which have excellent conformational dynamics leading towards strong binding affinity to cancerous cells [8,9,10,26]. In this present study, we have tailored spherical GNPs (~4 nm) with a T1 gadolinium-based contrast agent (Gd(III)-DO3A-SH) and therapeutic/cancer-targeting DNA aptamer (AS1411) for cancer imaging and therapy. The GNP-Gd(III)-DO3A-SH-AS1411 probes were characterized via UV–Vis spectroscopy, dynamic light scattering (DLS), transmission electron microscopy(TEM), zeta potential, dark field microscopy, energy-dispersive X-ray analysis (EDAX), and compared to their noncancer-targeted c-rich oligonucleotide (CRO) analogs and control (CTR) GNP-Gd(III)-DO3A-SH-CRO/CTR.

GBCA efficacy can also be improved on the back-end of the imaging process through use of image analysis algorithms. State-of-the-art image analysis methods for maximizing three-dimensional (3D) gadolinium enhanced MR contrast agents are intrinsically dependent on variations in the voxel volume (mm^3^) [36,37,38]. Additionally, the GBCA’s longitudinal T1 mapping is also maximized using algorithms with global analysis [39,40,41] and local voxel value analysis [37,38] followed by voxel-wise normalization [42] employing control anatomical structures. The above methods can greatly augment conventional methods for obtaining superior contrast in the longitudinal T1-Weighted (T1-W) MRI images, such as variations in slice thickness, field of view (FOV), image acquisition (repetition TR/ echo TE) time and acquisition matrix [43,44,45]. 

To accurately quantify the suitability of any targeted contrast agent in a non-invasive way requires accurate quantification of T1 relaxation differences between cancer cells and healthy cells. Current image-based analysis techniques are based on the difference in the mean T1-weighted signal intensity between the Region of Interest (ROI) containing test (with targeted contrast agent) and control (without targeted contrast agent) cells. The main limitations of the current image analysis approaches are that they do not have the ability to demonstrate local changes (voxel-based changes) in relaxation, nor can they account for local differences in T1 relaxation due to heterogeneity of the tissue as opposed to the contrast agent. Moreover, the current approaches rely on manually delineated, two-dimensional ROIs without taking advantage of the three-dimensional information of the T1-weighted MRI data. To overcome these limitations, we have developed a new algorithm that has the ability to automatically extract the Volume of Interest (VOI) and apply 3D nonrigid registration to provide voxel-on-voxel matching that will aid in visualizing the T1 relaxation locally using a color map, as well as calculating global contrast agent-induced changes in relaxation. The efficacy of the algorithm is demonstrated using GBCAs conjugated to cancer-targeting gold nanoparticle/DNA aptamer constructs.

## 2. Materials and Methods

### 2.1. Materials

Gold (III) Chloride Trihydrate (HAuCl_4_·3H_2_O) was purchased from Alfa Aesar (Tewksbury, MA, USA). Citric acid, trisodium salt (Na_3_C_6_H_5_O_7_), sodium borohydride (NaBH_4_), gadolinium trichloride hexahydrate (GdCl_3_·6H_2_O), dithiothreitol (DTT), and anhydrous sodium bicarbonate (NaHCO_3_) were purchased from Sigma Aldrich (St. Louis, MO, USA). 10.0× PBS (pH 7.4) and Oligreen dye were purchased from Thermo Fisher Scientific (Waltham, MA, USA). Nanopure ultrapure water (Barnstead, resistivity of 18.2 MΩ-cm) was used for preparing all aqueous solutions. Hydrochloric acid (HCl) and Nitric Acid (HNO_3_) were analytical grades and purchased from VWR (Rednor, PA). Aqua regia solution (3 parts HCl and 1 part HNO_3_) was used to clean all glassware for GNP synthesis as well as the Xylenol Orange assay for Gd content. The tetraazamacrocycle 10-(2-sulfanylethyl)-1,4,7,10-tetraazacyclododecane-1,4,7-triacetic acid (H_4_DO3ASH) was purchased from Macrocyclics, Inc. (Dallas, TX). Oligonucleotides having a regular DNA backbone (phosphodiester), a 5’-Thiol C6 S-S modification (Thio-MC6-D), 5’-6T spacer (for AS1411 and CRO) and high-performance liquid chromatography (HPLC) purification were supplied by Integrated DNA Technologies (IDT) [Coralville, IA]. The oligonucleotides sequences used were 5’TTTTTTGGTGGTGGTGGTTGTGGTGGTGGTGGTTT (AS1411), 5’-TTTTTTCCTCCTCCT CCTTCTCCTCCTCCTCCTTT (CRO) and TTTTTT (CTR). The MDA-MB-231 breast cancer cells and MCF-10A healthy mammary gland cells were purchased from ATCC (Manassas, VA, USA). MEBM media with Bullet Kit Media were purchased from Lonza Group (Basel, Switzerland). Dulbecco’s Modified Eagle’s Medium (DMEM), fetal bovine serum (FBS), 10× Trypsin, TrypLE cell dissociation enzymes, penicillin–streptomycin (Pen/Strep), and Gentamicin were all purchased from Thermo Fisher Scientific (Waltham, MA, USA). Amicon Ultra 15.0 mL centrifugal filters with Ultracel-30 (30,000 MWCO) were purchased from Merck Millipore (Billerica, MA, USA) to remove excess oligonucleotide and Gd*(III)*-DO3A-SH, respectively. The 0.6 mL Eppendorf microcentrifuge tubes were purchased from Sigma Aldrich (St. Louis, MO, USA) to mount samples at 9.4 T (Agilent) MRI scanner. 

UV absorption spectra were measured with the UV Visible Spectrometer (Varian Cary 50 BIO UV, Agilent Technologies, Santa Clara, CA, USA). Dynamic Light Scattering measurements (DLS) were acquired on a Zetasizer (Zetasizer Nano ZS90, Malvern Instruments Ltd., Westborough, MA, USA). The Zeta potential measurements were acquired on latter samples using a NanoBrook Zeta PALS Zeta Potential Analyzer (Brookhaven Instruments, Holtsville, NY, USA). Tunneling electron microscopy (TEM) studies were performed on the FEI Tecnai F20 TEM to determine GNP morphology and distribution. Fluorescence studies were performed on an enhanced dark-field fluorescence microscope (CytoViva, Inc., Auburn, AL, USA).

### 2.2. Chemical Synthesis of 4-nm Gold Nanoparticles (GNPs)

Gold nanoparticles were synthesized according to the procedure described by Murphy and coworkers [46,47]. A volume of 2.5 mL of 0.01 M citric acid, trisodium salt (TRIS) was added to 95.0 mL of nanopure water under intense stirring. Then, 2.5 mL of the 0.01 M HAuCl_4_ solution was added followed immediately by 3.0 mL of 4 °C 0.1 M sodium borohydride. The solution was stirred for 2 h. GNP size was determined by UV–Vis and dynamic light scattering [48].

### 2.3. Chemical Synthesis of Gadolinium (III) DO3A-SH (Gd(III)-DO3A-SH)

The tetraazamacrocycle 10-(2-sulfanylethyl)-1,4,7,10-tetraazacyclododecane-1,4,7-triacetic acid (DO*3*A-SH) was chelated with GdCl_3_ by mixing an aqueous solution of DO*3*A-SH (15.8 µmol; 100 μL) with an aqueous solution of GdCl_3_ (347 mM; 50 µL) at room temperature [49,50,51]. The reaction mixture pH was then adjusted to 6.0 with 1.0 M sodium bicarbonate and the mixture incubated overnight at 60 °C. During the reaction, the pH was measured three times and bicarbonate solution added as necessary to keep the pH in the range of 6 to 7. Afterwards, the reaction mixture pH was adjusted to 9–10 using sodium bicarbonate and then centrifuged at 3000× *g* for 15 min. The sample was then vacuum filtered and the precipitate air-dried. 

### 2.4. Preparation of AS1411/CRO/CTR for Conjugation to GNP

Oligonucleotides having a regular DNA backbone (phosphodiester), a 5’-Thiol C6 S-S modification (Thio-MC6-D), and that were HPLC purified were supplied by Integrated DNA Technologies (IDT). Oligonucleotide solutions were prepared as previously reported [52]. Briefly, 500.0 µL of 500 µM oligonucleotide solution was prepared by suspending AS1411/CRO/CTR in nanopure water. Prior to use, the disulfide protecting group on the oligonucleotide solution was cleaved with dithiothreitol (DTT). A 250.0 µL solution of 1 M DTT was added to 60.0 µL of 500 µM oligonucleotide solution and heated at 90 °C for 1 h (0.1 M DTT, 0.18 M phosphate buffer (PB), pH 8.0). The cleaved oligonucleotides were purified using a NAP-5 column eluted with PB. Thereafter, the eluted solution of freshly cleaved oligonucleotides was added to gold nanoparticle dispersions.

### 2.5. Chemical Synthesis of GNP-Gd(III)-DO3A-SH-Oligonucleotide

Gold nanoparticles (GNP) were functionalized with the MRI contrast agent Gd(*III*)-DO*3*A-SH and/or thiol-deprotected oligonucleotide (AS1411/CRO/CTR). The freshly cleaved oligonucleotide solution was added to the GNP dispersion (~170 nM) [47] to bring the mixture to 1.2 µM oligonucleotide concentration [52], while simultaneously adding Gd(III)-DO3A-SH to achieve 1.3 mM contrast agent concentration. The GNP-Gd(III)-DO3A-SH-Oligonucleotide solution was incubated at room temperature for 20 min and then sonicated for 10 min. The solution was incubated overnight at 37 °C. The concentration of NaCl was gradually increased to 137 mM using 10× PBS over 3 days. During each salt addition, the GNP-Gd(III)-DO3A-SH-Oligonucleotide solution was sonicated for 60 s followed by incubation at 37 °C. As mentioned, the excess oligonucleotide and Gd(III)-DO3A-SH were removed via centrifugal filtration at 3000× *g* for 30 min. The concentrated solution was resuspended to 15.0 mL with 1.0× PBS and filtered two more times before use. 

### 2.6. Oligonucleotides Quantification

Forty-five microliters of the centrifuged pellets of GNP-Gd(III)-DO3A-SH-Oligonucleotide was redispersed to 2.0 mL samples at 45× dilution (5 μL of 1.0 M DTT, 0.1 M phosphate buffer (pH 8)) and incubated overnight at 37 °C to cleave the gold oligonucleotide–thiol bond. Within 24 h, after the gold nanoparticles precipitated, the supernatant was collected and released oligonucleotides were quantified using UV absorption spectra.

### 2.7. Transmission Electron Microscopy

Transmission electron microscopy (TEM) images were obtained using a FEI Tecnai F20 TEM. A field emission gun (FEG) was used for the electron source and the studies were performed with an accelerating voltage of 360 keV. The samples were prepared by suspending 20.0 μL of 0.5 to 0.6 μM concentration of GNP samples onto a C-flat holey carbon film mesh on copper grids and air dried overnight. A size histogram analysis of the TEM images was performed with ImageJ software (Ver 1.3, Bethesda, MD, USA).

### 2.8. Confirmation of Oligonucleotide Coating via Fluorescence

The samples were tagged with the fluorescent dye Oligreen™ using the protocol provided by the manufacturer. Excess dye was removed through centrifugation at 3000× *g* for 30 min. A Modulus Fluorimeter (Promega, Madison, WI) was used to measure the fluorescence of the samples (Blue module: Ex 460 nm, Em: 518–570 nm). The samples were also imaged with enhanced dark-field (CytoViva) fluorescence microscope. The samples were prepared by drop casting 10.0 μL of sample onto a glass microscope slide (Fisherbrand Superfrost Plus) and then placing a coverslip overtop (VWR microcover glass). The lacquer (Nail Polish) was then used to create a waterproof seal around the edges. The samples were imaged at 60× magnification.

### 2.9. Gadolinium Quantification with Energy-Dispersive X-Ray Analysis (EDAX)

EDAX was performed on a Zeiss SUPRA 35 FE-SEM (Zeiss Peabody, MA) at 20 KeV incident beam at 8.1 mm working distance. The atomic ratios of Gd:Au were measured for GNP-Gd(III)-DO3A-SH-oligonucleotide to quantify Gd content per GNP. 

### 2.10. Gadolinium Quantification with Xylenol Orange Titration

Gadolinium quantification was performed by monitoring relative changes in the UV–Vis absorption bands (434 nm and 573 nm) of xylenol orange upon complexation with free Gd^3+^ ions using the calibration equation Y = 0.3224X − 1.0944 [53,54]. An aliquot of GNP-Gd(III)-DO3A-SH Oligonucleotide (Concentration; Volume: 30 µM; 70.0 µL) was diluted with 430.0 µL aqua regia solution and heated at 80 °C for 24 hours to release free Gd(III) ions into solution [53]. The pH was adjusted to 6.0 using 1.0 M NaOH and diluted to total volume of 2.0 mL with acetate buffer (pH 5.8). A 50 µL aliquot of this pH-adjusted solution was added to 1950.0 µL of xylenol orange solution and the ratio of A_573_/A_433_ was recorded for quantification of free Gd^3+^ ions using Equations (1) and (2) [54].

(1) $$Gd\left(III\right)\propto Abs\left(\frac{573}{433}\right)nm$$

(2) $$\left[Gd\left(III\right)\right]=\left[A+B\times Abs\;\left(\frac{573}{433}\right)\right]$$

The coefficients A = 3.0826 and B = 1.0944 of Equation (2) were retrieved from the free Gd(III) calibration equation Y = 0.3244X − 1.0944. 

### 2.11. Cell Culture

Breast cancer cells (MDA-MB-231) and nonmalignant breast epithelial cells (MCF-10A) were obtained from ATCC (Manassas, VA, USA). The MDA-MB-231 and MCF-10A cells were maintained in Dulbecco’s Modified Eagle’s Medium (DMEM) and Mammary Epithelial Cell Basal Medium (MEBM), respectively. Cells were cultured in a 5% CO_2_ incubator at 37 °C. 

### 2.12. Magnetic Resonance Imaging (MRI) Agarose Phantoms

Samples for MRI imaging were prepared by mixing different concentrations (1200, 300, and 75 nM) of gold nanoparticles functionalized with Gd(III)-DO3A-SH and oligonucleotide (AS1411/CRO/CTR) with agarose solution (0.7%, 55 °C) in 1:1 ratio in 0.5 mL Eppendorf microcentrifuge tubes. The uniform dispersions were allowed to stand at room temperature for 30 min to solidify. Samples were mounted on a mesh grid and MRI images were acquired on the 9.4 T instrument (Agilent, Santa Clara, CA, USA) for T1-W and T1 MRI measurements. 

### 2.13. Magnetic Resonance (MR) Imaging

All MR imaging data were acquired using an Agilent 9.4 T horizontal bore MRI system equipped with Agilent 205/120 HD gradient coil (Agilent Technologies, Santa Clara, CA, USA). A RAPID 72-mm volume coil was used for signal transmission and detection (RAPID MR International, Columbus, OH, USA). After a quick 3-plane gradient echo-based scout scan was performed to verify sample positioning, T1-W images were obtained using a standard spin echo multislice (SEMS) imaging sequence with the following parameters: TR/TE = 500/10 msec; matrix size = 128 × 128; FOV = 40 × 40 mm^2^; 13 slices with a slice thickness of 1.0 mm. When acquiring data for T1 calculation purposes, a fast spin echo multislice (fSEMS) sequence was used with the following parameters: TR (array) = 6000, 4700, 3600, 2800, 2200, 1700, 1300, 1000, 800 msec; effective TE = 20 msec; matrix size = 128 × 128; FOV = 40 × 40 mm^2^; 9 slices with a slice thickness of 2.0 mm and 2 averages. All data from the 9.4T MRI system were converted to Digital Imaging and Communications in Medicine (DICOM) format using Varian NMR J (VNMRJ) 4.0 interface software (Palo Alto, CA, USA) before transferring to other software for further data processing. Samples were also scanned using a 3 T Siemens MAGNETOM Skyra (Siemens Medical Solutions, Malvern, PA, USA) with a 20-channel head coil. A turbo spin echo (TSE) sequence was used under 3 T for T1 quantification: TR (array) = 5000, 2000, 1000, 750, 500 msec; TE = 10 msec; matrix size = 256 × 256; FOV = 18 × 18 cm^2^; 17 slices with a slice thickness of 1.4 mm.

### 2.14. Determination of GNP-Gd(III)-DO3A-SH-Oligonucleotide Cellular Uptake Using Live MDA-MB-231 and MCF-10A

MDA-MB-231 and MCF-10A were then treated with different concentrations of gold nanoparticles (1200 nM, 300 nM, and 75 nM) functionalized with Gd(III)-DO3A-SH and AS1411/CRO/CTR made in complete DMEM and MEBM, respectively, and incubated for 24, 48, 72, and 96 h time points at 37 °C to allow cell uptake and localization. After the prescribed incubation time, the media was discarded and cells were washed 3 times with 1× PBS to remove any excess and unbound gold nanoparticles that were not internalized within the cells. Cells were detached from the monolayer using TrypLE cell dissociation enzyme solution for 5 min at 37 °C, transferred to a 15 mL conical tube, and centrifuged at 700 rpm to pellet the cells and separate any remaining unbound gold nanoparticles. Excess gold nanoparticles and TrypLE solution were removed and cell pellets were suspended in 100.0 μL of 1× PBS, and transferred to 0.5 mL tubes with the addition of 100.0 µL of 1.4% agarose to give a final concentration of 0.7% agarose for a clear homogenous solution. Cells were then mounted on the 9.4 T instrument mesh grid holder for T1-W and T1 MRI measurements.

### 2.15. T1-Weighted Image Analysis Algorithm

The T1-Weighted image calculation algorithm starts with selection of the two Eppendorf microcentrifuge tubes (0.5 mL) to be analyzed (see Figure S1a). Then, a background removal and 3D region-growing algorithm was applied to extract the VOI that contained each eppendorf microcentrifuge tube in the middle of the VOI (see Figure S1b–f). The second step was to perform a two-step 3D registration technique to guarantee a voxel-on-voxel match between the sample and control for accurate reflectivity calculations. The registration technique began with a global alignment using a 3D affine transformation model. In order to perform the global registration, the distance maps inside the reference and target Eppendorf microcentrifuge tubes were generated by finding the minimum Euclidean distance for every inner voxel to the object boundary using a fast marching level set. Subsequently, a 3D affine transformation with 12 degrees of freedom was applied to align the target Eppendorf microcentrifuge tube to the reference tube by maximizing the mutual information of the generated distance maps. The second registration step was to perform a nonrigid alignment (a step confirming voxel-on-voxel match) using a multiresolution elastic registration approach that was based on 3D cubic B-splines with four-stage multiresolution together with limited memory Broyden–Fletcher–Goldfarb–Shanno (L-BFGS-B) optimization [55]. Example results to demonstrate this registration approach are shown in Figure S2. 

The traditional method to calculate the reflectivity is as follows

(3) $$\mathrm{Reflectivity}\left(R\right)=\frac{{\left(\mathrm{Test}\right)}_{\mathrm{avr}}-{\left(\mathrm{Control}\right)}_{\mathrm{avr}}}{{\left(\mathrm{Control}\right)}_{\mathrm{avr}}}\times 100$$

**Figure 1:**
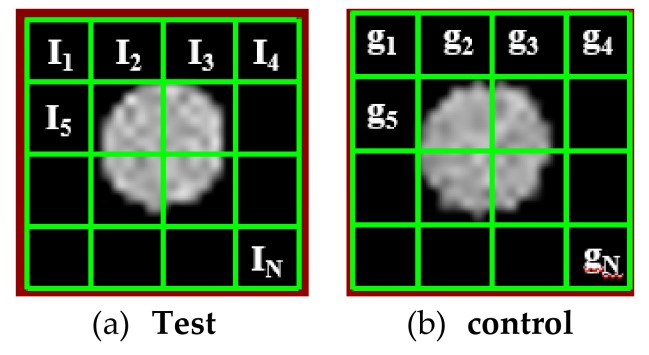
Illustration of the reflectivity calculation using (**a**) the segmented image from the test sample MRI and (**b**) the segmented area of the control sample MRI image.

**Figure 2:**
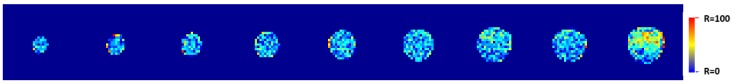
An illustration of the color map generated by the MRI analysis algorithm.

Since after aligning the MRI data, we have voxel-on-voxel match, thus the reflectivity was calculated as follows

(4) $$\mathrm{Reflectivity}\left(R\right)=\frac{\sum_{i=1}^{N}{\left(I_{i}\right)}_{\mathrm{test}}-{\left(g_{i}\right)}_{\mathrm{control}}}{\sum_{i=1}^{N}{\left(g_{i}\right)}_{\mathrm{control}}}\times 100$$

where N was the number of voxels in each image, I_i_ was the MRI signal of the test cells and g_i_ was the MRI signal of the control cells as demonstrated in Figure 1. Note that each MR scan had one Eppendorf microcentrifuge tube filled with water. All of the data from any MRI scan was divided by the average signal of water. Finally, after calculating the reflectivity using Equation (4), the approach generated a color map that showed the reflectivity of each voxel as shown in Figure 2.

### 2.16. Statistical Comparison of VNMRJ and Algorithm Contrast Measurements

Paired *t*-tests (GraphPad prism version 6.00, GraphPad software, San Diego, CA) were used to analyze the statistical significance for differences in ROI-based percent contrast enhancement measurements for both algorithm and 9.4 T VNMRJ (Agilent Technologies, Santa Clara, CA, USA) analyses. Paired *t*-tests (parametric, two-tailed) were used to compare algorithm results with VNMRJ software for all samples in MDA-MB-231 and MCF-10A cells. All reported data represents the mean values and standard errors of the mean. Significance was considered to be present at *p* < 0.05 for all data.

## 3. Results and Discussion

Citrate capped gold nanoparticles (GNP) were synthesized via NaBH_4_ reduction method [47] and subsequently functionalized with the MRI contrast agent Gd(III)-DO3A-SH [50] and/or thiol-deprotected oligonucleotide (AS1411/CRO/CTR) [52], respectively. Table 1 contains DLS, TEM, and zeta potential characterization of the solutions of citrate capped GNP and GNP-Gd(III)-DO3A-SH Oligonucleotide in conjunction with quantification of the functionalization agents Gd(III)-DO3A-SH and AS1411/CRO, respectively. A zeta (ζ) potential of −58–20 mV was observed for GNP-Gd(III)-DO3A-SH Oligonucleotide solutions, which displayed high stability and resistance to aggregation in PBS dispersions. On the other hand, citrate capped GNP aggregated in PBS dispersions; therefore, the zeta (ζ) potential, hydrodynamic size and TEM characterizations were performed in nanopure water. Citrate-capped GNP and GNP-Gd(III)-DO3A-SH-Oligonucleotide solutions were stable for more than six months in solution at 4 °C and 37 °C, respectively. 

**Figure 3:**
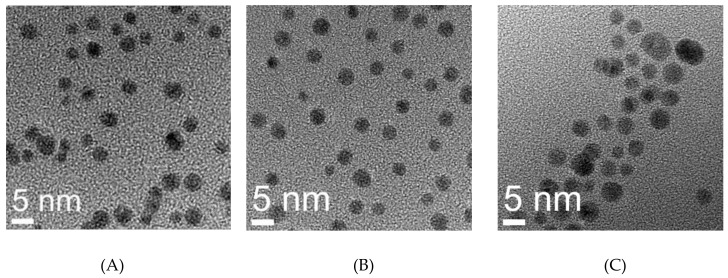
TEM images of (**A**) GNP-Gd-DO3A-SH-AS1411, (**B**) GNP-Gd-DO3A-SH-CRO, and (**C**) GNP-Gd-DO3A-SH-CTR at 360,000× magnification.

**Figure 4:**
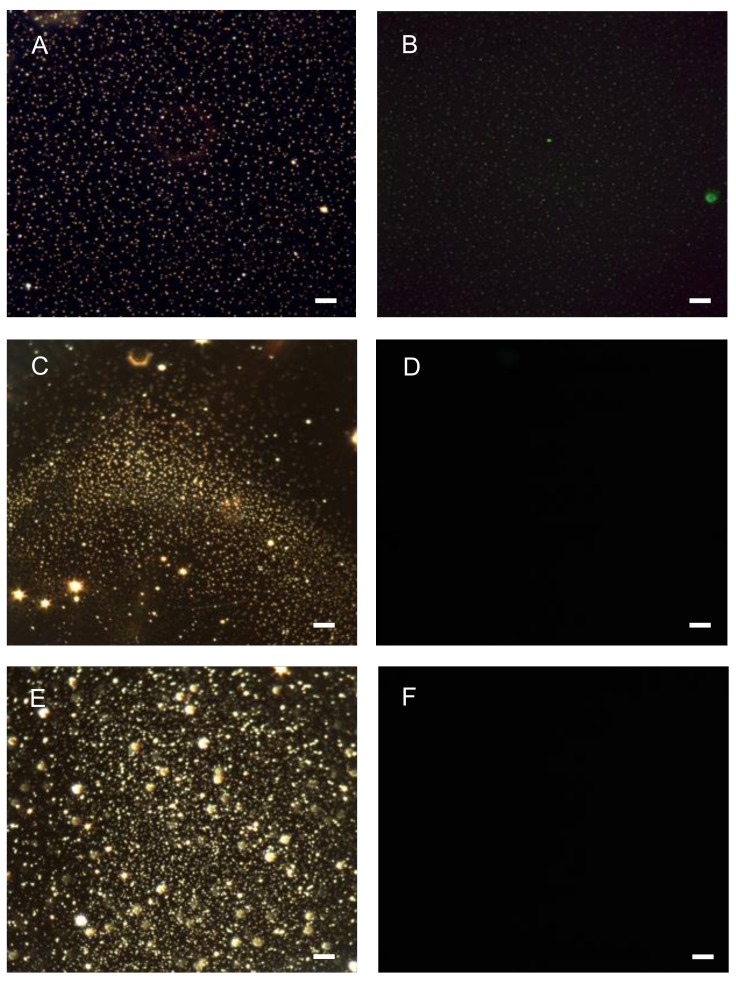
(**A**) Dark-field and (**B**) fluorescence images of OliGreen® tagged GNP-Gd(III) DO3A-SH-AS141. (**C**) Dark-field and (**D**) fluorescence images of GNP-Gd(III)-DO3A-SH and (**E**) Dark-field and (**F**) fluorescence images of non-OliGreen® tagged GNP-AS1411. Scale bar = 10 microns.

The compact monomeric quadruplex conformation of AS1411 leads to the relatively smaller hydrodynamic diameter of GNP-Gd(III)-DO3A-SH-AS1411 in comparison to GNP-Gd(III)-DO3A-SH-CRO, Table 1. TEM imaging of GNP-Gd(III)-DO3A-SH-Oligonucleotide (AS1411/CRO/CTR) revealed average gold core diameters of 3–6 nm, Figure 3. The AS1411 oligonucleotide was detected on the GNP surface using Quant-iTTM OliGreen® single-stranded DNA binding reagent via fluorescence studies on the 6–8 µM purified pellets of GNP-Gd(III)-DO3A-SH AS1411 and GNP-Gd(III)-DO3A-SH, Figure 4 and Table S1. 

**Figure 5:**
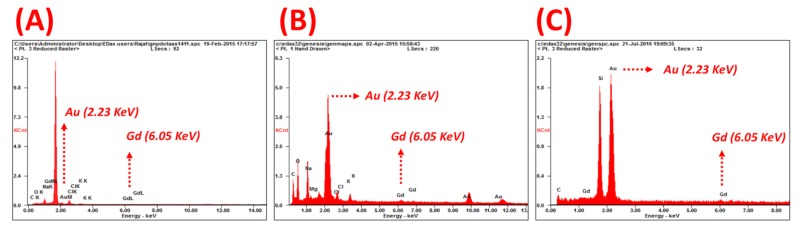
EDAX spectra of (**A**) GNP-Gd(III)-DO3A-SH-AS1411, (**B**) GNP-Gd(III)-DO3A-SH-CRO, and (**C**) GNP-Gd(III)-DO3A-SH-CTR collected with 20 KeV incident beam and displaying gold (2.23 KeV) and gadolinium (6.05 KeV) peaks for gadolinium (Gd) quantification from Gd:Au atomic ratios.

The gadolinium content per GNP, Table 1, was quantified using both a Xylenol Orange gadolinium quantification assay and EDAX analysis techniques [53,54] (Tables S2–S6, respectively). The EDAX analysis of GNP-Gd(III)-DO3A-SH-oligonucleotide, yielded reliable energy peaks for gold (Au) and gadolinium (Gd) at 2.23 KeV and 6.05 KeV, respectively, Figure 5. The atomic ratios of Gd:Au were subsequently measured to quantify Gd content per GNP, Tables S2–S5. These values were used in the MRI studies to match the Gd content in the nanoparticle samples to the samples of Multihance, a clinically utilized GBCA that serves as a positive control in our study. Both AS1411 and Gd(III)-DO3A-SH contain a terminal thiol (-SH) group to allow facile binding to the gold nanoparticle surface as a mixed coating [3,30,52]. AS1411 was included in the coating to allow the nanoparticles to be selectively retained in the cancer cells rather than healthy cells. 

To evaluate the selective targeting-induced [8,9] contrast enhancement [30,32,56] by GNP-Gd(III)-DO3A-SH-Oligonucleotide, longitudinal T1 relaxation and T1-W measurements were made at nanomolar GNP concentrations (1200, 300, and 75 nM) and compared with corresponding clinically relevant Multihance concentrations (1700, 7000, and 27,900 nM) in triple-negative breast cancer cells (MDA-MB-231) and nonmalignant breast epithelial cells (MCF-10A), Table 2, Table 3 and Table 4. In each table, the amount of contrast enhancement is listed for each concentration of the various contrast agents as calculated by either VNMRJ (Table 2 and Table 3) or our algorithm (Table 4). This allows a direct viewing of the concentration dependence of the contrast enhancement and also allows comparison between samples and contrast measurement techniques. While 9.4 T MRI uses a higher magnetic field than currently used in the clinic, the technique has been shown to be safe for in vitro use for up to five days of continuous scanning without effects on cell growth and differentiation [57]. Since our scanning is completed in 15–20 min, we do not expect any adverse effects from the MRI scans to complicate our results. Samples of GNP-Gd(III)-DO3A-SH-AS1411/CRO in 1.0× PBS solution displayed higher relaxivity values at 9.4 T compared to GNP-Gd(III)-DO3A-SH-CTR, Table 5 and Figures S3–S5. Overall, these marked increases in relaxivity values were noted for the gadolinium chelates co-coated onto gold nanoparticles with longer sequence oligonucleotides (AS1411/CRO) in comparison to their shorter sequence counterpart (CTR) or the free chelate. This higher relaxivity/T1-W contrast of GNP-Gd(III)-DO3A-SH-AS1411/CRO in comparison to GNP-Gd(III)-DO3A-SH-CTR was attributed to the slower rotational tumbling rate of the paramagnetic Gd(III)-DO3A-SH in the GNP-Gd(III)-DO3A-SH-AS1411/CRO agent. The slower rotational tumbling rate of the Gd(III) center was attributed to steric bulk (noncovalent interactions; steric hindrance) [58] generated by the more bulky 35-mer oligonucleotides (AS1411 and CRO) in comparison to the six base pair oligonucleotide (CTR) on the surface of the rigid GNP core (~4 nm) shell. Moreover, the water protons relaxation was primarily due to the inner sphere chemical exchange because of the steric hindrance by the oligonucleotides impeding outer sphere exchange [23,59]. 

Though the traditional T1-W images involved a rapid acquisition time (TR and TE), they possessed irreproducible intensity profiles in comparison to the T1 maps. This was due to the T1-W intensities being dependent upon instrumental parameters, in contrast to T1 maps that were a measure of the inherent spin-lattice relaxation times of the sample. However, by employing our algorithm using the optimized T1-W image parameters (as outlined in experimental Section 2.15), GNP-Gd(III)-DO3A-SH-Oligonucleotide exhibited enhanced, rapid, sensitive and more reproducible longitudinal T1-W image analysis compared to VNMRJ ROI analysis of the T1 images at each time point (24–96) h using 3 × 10^7^ breast cancer cells (MDA-MB-231) and 3 × 10^7^ nonmalignant breast epithelial cells (MCF-10A). 

The local voxel and global image analysis on the GNP-Gd(III)-DO3A-SH-Oligonucleotide construct showed exceptional contrast enhancement for MDA-MD-231 cells targeted by the cancer specific contrast agent GNP-Gd(III)-DO3A-SH-AS1411 compared to its nontargeted control counterparts GNP-Gd(III)-DO3A-SH-CRO/CTR and Multihance. While both the new T1-W image analysis algorithm and the VNMRJ-based ROI analysis yielded considerable T1-W contrast enhancement for both targeted GNP-Gd(III)-DO3A-SH-AS1411 and nontargeted GNP-Gd(III)-DO3A-SH CRO/CTR contrast agents at sub-millimolar concentrations, the VNMRJ-based ROI analysis of T1-W and T1 images was unable to differentiate selective targeting-induced contrast enhancement of the targeted GNP-Gd(III)-DO3A-SH-AS1411 from nontargeted GNP-Gd(III)-DO3A-SH-CRO/CTR (Table 2, Table 3 and Table 4). On the other hand, our new T1-W image analysis algorithm displayed a higher magnitude of T1-W contrast with targeted GNP-Gd(III)-DO3A-SH-AS1411, depicting enhanced uptake [8,25] of the targeted oligonucleotide (AS1411) conjugated contrast agent in malignant MDA-MD-231 cells compared to nontargeted control counterparts GNP-Gd(III)-DO3A-SH-CRO/CTR. 

**Figure 6:**
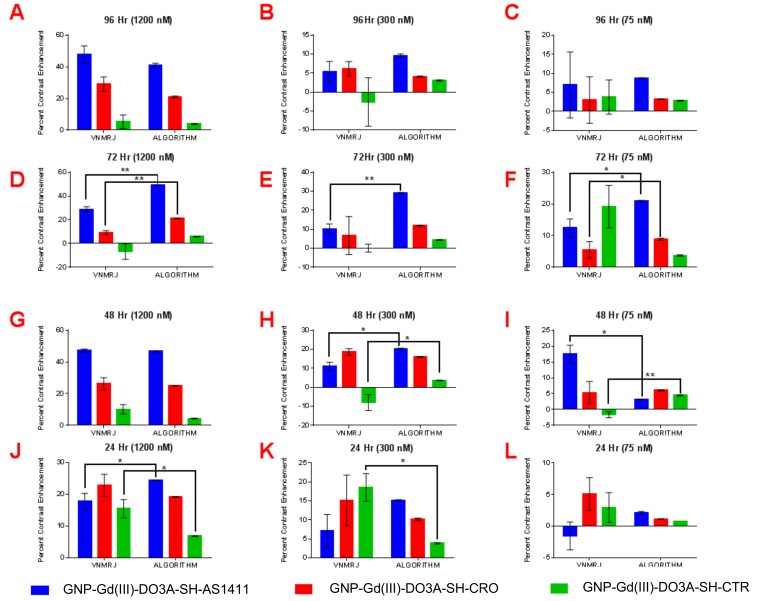
Paired *t*-test analysis of percent contrast enhancement: VNMRJ vs. algorithm in MDA-MB-231 cell lines using region of interest analysis (*n* = 3) at the following time points/GNP-Gd(III)-DO3A-SH-Oligonucleotide concentrations; (**A**) 96 h/1200 nM, (**B**) 96 h/300 nM, (**C**) 96 h/75 nM, (**D**) 72 h/1200 nM, (**E**) 96 h/300 nM, (**F**) 72 h/75 nM, (**G**) 48 h/1200 nM, (**H**) 48 h/300 nM, (**I**) 48 h/75 nM, (**J**) 24 h/1200 nM, (**K**) 24 h/300 nM, and (**L**) 24 h/75 nM. Two-tail *p*-values (95% CI): * < 0.05; ** < 0.009.

**Figure 7:**
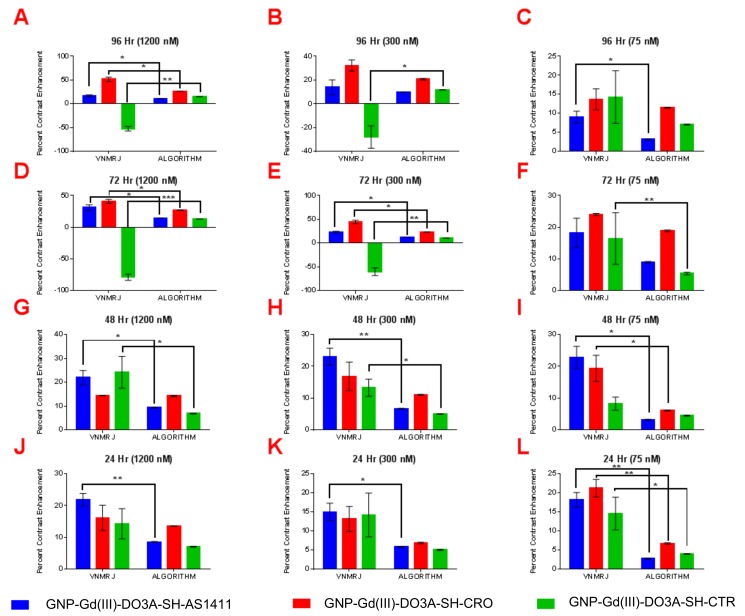
Paired *t*-test analysis of percent contrast enhancement: VNMRJ vs. algorithm in MCF-10A cell lines using region of interest analysis (*n* = 3) at the following time points/GNP-Gd(III)-DO3A-SH oligonucleotide concentrations; (**A**) 96 h/1200 nM, (**B**) 96 h/300 nM, (**C**) 96 h/75 nM, (**D**) 72 h/1200 nM, (**E**) 96 h/300 nM, (**F**) 72 h/75 nM, (**G**) 48 h/1200 nM, (**H**) 48 h/300 nM, (**I**) 48 h/75 nM, (**J**) 24 h/1200 nM, (**K**) 24 h/300 nM, and (**L**) 24 h/75 nM. Two-tail *p*-values (95% CI): * < 0.05; ** < 0.009; *** = 0.0009.

The observed higher T1-W contrast of GNP-Gd(III)-DO3A-SH-AS1411, in comparison to GNP-Gd(III)-DO3A-SH-CRO/CTR, was due to enhanced accumulation of the AS1411-coated GNPs in the breast cancer cells (MDA-MB-231) [8]. The AS1411 oligonucleotide had unique conformational dynamics and binding affinity in the breast cancer (MDA-MB-231) cell lines. Though the AS1411 oligonucleotide had a regular DNA backbone (phosphodiester), 5’-Thiol C6 S-S modification (Thio-MC6-D), it was known to form G-quadruplex structures that selectively bound to the protein nucleolin [10]. Although nucleolin was found solely in the nucleus of the healthy cells, non-nuclear nucleolin proteins are present in the cytoplasm and the plasma membrane of most cancer cells [8,9,10]. While non-nuclear nucleolin seemed to facilitate the survival of cancer cells via increasing the concentration of antiapoptotic mRNA and miRNA in the cytoplasm (amongst other confirmed functions), it also provided a convenient target for nanoparticle constructs coated with AS1411 as a means to target cancer cells. As non-nuclear nucleolin proteins were not present in the cytoplasm of nonmalignant breast epithelial cells (MCF-10A), the nucleolin-targeted oligonucleotide (AS1411) conjugated contrast agent GNP-Gd(III)-DO3A-SH-AS1411, as well as nontargeted control counterparts GNP Gd(III)-DO3A-SH-CRO/CTR, had lower accumulation in MCF-10A cells, and thus displayed lower T1-W contrast values. The reproducibility of contrast measurements using VNMRJ ROI selection vs. our algorithm was also examined, see Figure 6 and Figure 7. Our algorithm yielded a similar MRI contrast enhancement (19.5% difference) compared to the VNMRJ software for micromolar concentrations of the contrast agents (41.2% vs. 51.2% enhancement, respectively). However, all contrast measurements performed using our algorithm showed a high degree of reproducibility, whereas ROI analysis using VNMRJ was widely inconsistent. Additionally, our algorithm produced significantly higher MRI contrast enhancement (~500% difference) compared to the VNMRJ software at nanomolar concentrations of the contrast agents (18.2 vs. 3.1% enhancement, respectively). Even though the contrast values determined using either technique were found to be similar in several cases, significant differences in contrast enhancement values were noted in 35 out of 72 samples measured. Therefore, it is anticipated that the added sensitivity and reproducibility of our MRI analysis algorithm may prove useful for in vivo studies where delivery of nanoparticle-based contrast agents to the tumor site is limited by poor pharmacokinetics. 

## 4. Conclusions

In this study, we have demonstrated the ability to functionalize gold nanoparticles (GNP-Gd(III)-DO3A-SH-) with the targeted oligonucleotide AS1411, which significantly increased the uptake of MRI imaging contrast agent into malignant MDA-MD-231 cells compared to nontargeted control counterparts. The cost to produce the GNP-Gd(III)-DO3A-SH-AS1411 is comparable to other imaging contrast agents currently in use. For example, it is estimated that this contrast agent will cost ~$2 per cell treatment. However, the bulk of the cost is not the gold nanoparticles themselves, rather the oligonucleotide, AS1411, is the most costly component of the contrast agent. In addition, this study successfully implemented a new local voxel-wise MRI analysis algorithm. This new algorithm was found to yield similar contrast image results as the current gold standard (VNMRJ software) at micromolar concentrations of the contrast agents; however, our algorithm significantly enhanced MRI image contrast at nanomolar concentrations.

## Figures and Tables

**Table 1:** Dynamic light scattering (DLS), TEM, and zeta potential characterization of citrate capped GNP and GNP Gd(III) DO3A-SH oligonucleotide and quantification of the functionalization agents Gd(III)-DO3A-SH and AS1411/CRO.

Sample	Hydrodynamic Size (nm)	TEM (nm)	Zeta Potential	Quantification of Functionalization Agents	
				* Gd	** Oligonucleotide
GNP-Gd(III)-DO3A-SH AS1411	13.5 ± 2.1	3.4 ± 0.6	−20.9 ± 1.1	23.23 ± 0.93	10 ± 2
GNP-Gd(III)-DO3A-SH CRO	19.3 ± 2.5	3.3 ± 0.6	−57.6 ± 1.0	19.21 ± 5.57	9 ± 2
GNP-Gd(III)-DO3A-SH CTR	9.83 ± 1.45	5.45 ± 1.42	−42.67 ± 4.15	13.95 ± 0.73	
GNP Citrate	4.88 ± 0.51	4.0 ± 0.7	−16.25 ± 2.30		

* Gadolinium quantification via Xylenol Orange Titration; ** Oligonucleotide quantification via 1.0 M Dithiothreitol (DTT) cleavage of gold oligonucleotide–thiol bond.

**Table 2:** T1 Contrast enhancement comparison for GNP-Gd(III)-DO3A-SH Oligonucleotide in MDA-MB-231 and MCF-10A cell lines using Region of Interest analysis at Varian NMR J (VNMRJ) software.

T1 Contrast Enhancement (VNMRJ: Region of Interest (ROI)Analysis)																
Time (Hours)	96				72				48				24			
Cell Lines (type) with GNP Concentration (nM)	^*^ GNP-Gd(III)-DO3A-SH-Oligonucleotide			^**^ M	^*^ GNP-Gd(III)-DO3A-SH-Oligonucleotide			^**^ M	^*^ GNP-Gd(III)-DO3A-SH-Oligonucleotide			^**^ M	^*^ GNP-Gd(III)-DO3A-SH-Oligonucleotide			^**^ M
	A	B	C		A	B	C		A	B	C		A	B	C	
MDA-MB-231 (1200 nM)	31.2	23.2	8.6	13.9	35.7	26.9	15.9	11.7	35.5	29	17.1	11.7	16.5	21.2	6.9	17.6
MDA-MB-231 (300 nM)	15.4	10.0	3.3	9.3	24.9	22.5	13.3	16.1	14.4	26.9	13.6	18.3	3.1	11.3	10.2	26.4
MDA-MB-231 (75 nM)	0.83	4.7	1.3	3.4	16.4	15.4	18.7	10.4	7.8	16.9	6.7	11.8	0.1	0.0	0.0	15.0
MCF-10A (1200 nM)	23.4	72.8	15.4		37.8	68.5	38.0		15.2	16.6	18.8		15.1	16.1	20.4	
MCF-10A (300 nM)	21.5	39.9	11.9		30.8	53.4	27.6		20.1	22.0	17.5		12.8	16.4	19.6	
MCF-10A (75 nM)	7.3	11.5	7.0		20.7	20.4	23.5		12.3	15.4	8.3		7.2	10.9	15.6	

* A = AS1411, B = CRO, C = CTR; ** M = Multihance.

**Table 3:** T1-Weighted contrast enhancement comparison for GNP-Gd(III)-DO3A-SH oligonucleotide in MDA-MB-231 & MCF-10A cell lines using 2D region of interest analysis (*n* = 5) in VNMRJ software.

T1-Weighted Contrast Enhancement (VNMRJ: Region of Interest (ROI) Analysis)																
Time (Hours)	96				72				48				24			
Cell Lines (type) with GNP Concentration (nM)	^*^ GNP-Gd(III)-DO3A-SH- Oligonucleotide			^**^ M	^*^ GNP-Gd(III)-DO3A-SH- Oligonucleotide			^**^ M	^*^ GNP-Gd(III)-DO3A-SH- Oligonucleotide			^**^ M	^*^ GNP-Gd(III)-DO3A-SH- Oligonucleotide			^**^ M
	A	B	C		A	B	C		A	B	C		A	B	C	
MDA-MB-231 (1200 nM)	51.2 ± 6.1	27.4 ± 4.0	6.3 ± 3.5	4.7 ± 6.7	30.1 ± 2.5	11.4 ± 3.6	−4.9 ± 6.7	12.7 ± 4.8	46.8 ± 1.7	27.8 ± 4.6	8.3 ± 4.4	5.1 ± 10.9	20.2 ± 5.1	25 ± 4.56	13.0 ± 4.2	9.9 ± 4.6
MDA-MB-231 (300 nM)	7.6 ± 3.6	4.1 ± 7.6	−1.3 ± 5.9	8.1 ± 3.5	6.9 ± 4.9	6.9 ± 7.2	1.7 ± 3.3	16.4 ± 2.8	6.9 ± 5.9	21.0 ± 3.5	−7.6 ± 3.0	4.6 ± 4.6	7.0 ± 3.2	15.1 ± 4.7	18.9 ± 4.6	10.2 ±6.4
MDA-MB-231 (75 nM)	6.9 ± 8.7	3.0 ± 6.1	3.7 ± 4.5	7.3 ± 6.1	10.4 ± 3.5	6.1 ± 4.9	20.7 ± 5.7	5.9 ± 1.8	8.9 ± 12.4	6.3 ± 3.2	−2.0 ± 2.7	8.4 ± 5.4	−2.2 ± 1.9	4.7 ± 1.9	0.3 ± 5.9	2.0 ± 4.4
MCF-10A (1200 nM)	21.3 ± 2.3	16.0 ± 4.8	16.0 ± 4.2		31.2 ± 4.2	41.7 ± 2.4	−83.33 ± 7.2		22.6 ± 3.8	17.6 ± 4.5	23.2 ± 9.3		19.4 ± 7.2	50.9 ± 3.5	−48.6 ± 6.2	
MCF-10A (300 nM)	13.7 ± 3.2	13.5 ± 2.4	15.7 ± 4.6		19.8 ± 4.9	46.8 ± 4.5	−59.1 ± 6.9		24.8 ± 3.2	18.8 ± 4.2	12.2 ± 4.5		15.7 ± 5.2	31.5 ± 5.8	−29.4 ± 7.2	
MCF-10A (75 nM)	18.2 ± 2.6	22.2 ± 2.2	18.0 ± 5.8		13.8 ± 6.9	24.3 ± 1.4	10.8 ± 9.9		22.8 ± 3.5	19.6 ± 3.1	6.9 ± 2.7		8.6 ± 1.2	13.0 ± 2.3	12.8 ± 5.9	

* A = AS1411; B = CRO; C = CTR; ** M = Multihance.

**Table 4:** T1-Weighted contrast enhancement comparison for GNP Gd(III)-DO3A-SH oligonucleotide in MDA-MB-231 & MCF-10A cell lines using T1-weighted image analysis algorithm.

T1-Weighted Contrast Enhancement (Algorithm)																
Time (Hours)	96				72				48				24			
Cell Lines (type) with GNP Concentration (nM)	^*^ GNP-Gd(III)-DO3A-SH- Oligonucleotide			^**^ M	^*^ GNP-Gd(III)-DO3A-SH- Oligonucleotide			^**^ M	^*^ GNP-Gd(III)-DO3A-SH- Oligonucleotide			^**^ M	^*^ GNP-Gd(III)-DO3A-SH- Oligonucleotide			^**^ M
	A	B	C		A	B	C		A	B	C		A	B	C	
MDA-MB-231 (1200 nM)	41.2	21.1	3.9	4.3	49.1	21.0	5.7	6.7	46.8	25.0	4.3	6.9	24.4	19.2	6.8	7.3
MDA-MB-231 (300 nM)	9.6	4.1	3.1	2.9	29.2	11.9	4.3	5.1	20.1	16.0	3.7	5.8	15.2	10.2	3.9	6.1
MDA-MB-231 (75 nM)	8.7	3.2	2.8	0.8	20.8	8.8	3.7	1.6	9.0	8.0	3.4	4.9	2.1	1.1	0.7	5.4
MCF-10A (1200 nM)	11.0	26.4	15.1		14.5	26.9	12.9		9.3	14.3	6.9		8.5	13.6	7.0	
MCF-10A (300 nM)	9.6	20.7	11.9		12.1	23.3	10.3		6.7	11.1	5.0		5.8	6.9	5.1	
MCF-10A (75 nM)		11.5	7.0		8.9	18.8	5.4		3.2	6.1	4.5		2.7	6.7	4.1	

* A = AS1411, B = CRO, C = CTR; ** M = Multihance.

**Table 5:** Relaxivity values for gold nanoparticles functionalized with Gd(III)-DO3A-SH and oligonucleotides AS1411/CRO.

Samples	Relaxivity (mM^−1^s^−1^) at 9.4 T
GNP-(Gd(III)-DO3-SH)-AS1411	24.83
GNP-(Gd(III)-DO3A-SH)-CRO	15.67
GNP-(Gd(III)-DO3A-SH)-CTR	8.38
GNP-(Gd(III)-DO3A-SH)	5.56
Gd(III)-DO3A-SH	2.29
Multihance (*gadobenate dimeglumine)*	3.75

## Supplementary Materials

The following are available online at https://www.mdpi.com/2079-4991/9/5/709/s1, Figure S1: Illustration results of the automatic extraction of VOI. Figure S2: Illustration results of the nonrigid registration step. Figure S3: Relaxivity Curve for GNP-Gd(III)-DO3A-SH-AS1411 in 0.7% agarose solution (9.4 T MRI scanner). Figure S4: Relaxivity Curve for GNP-Gd(III)-DO3A-SH-CRO in 0.7% agarose solution (9.4 T MRI scanner). Figure S5: Relaxivity Curve for GNP-Gd(III)-DO3A-SH-CRO in 0.7% agarose solution (9.4 T MRI scanner). Table S1: Oligreen staining fluorescence values (FL) obtained for GNP Samples using Blue Module (Excitation 460 nm; Emission 515–570 nm). Table S2: Gadolinium quantification per gold nanoparticle in GNP-Gd(IIII)-DO3A-SH-Oligonucleotide. Table S3: Atomic and Weight percentages of elements analyzed in EDAX of GNP-Gd(III)-DO3A-SH-AS1411. Table S4: Atomic and Weight percentages of elements analyzed in EDAX of GNP-Gd(III)-DO3A-SH-CRO. Table S5: Atomic and Weight percentages of elements analyzed in EDAX of GNP-Gd(III)-DO3A-SH-CTR. Table S6: Gadolinium Quantification using xylenol orange Gd(III) protocol.
