# Supplementary material for: Targeted Gold Nanoparticle–Oligonucleotide Contrast Agents in Combination with a New Local Voxel-Wise MRI Analysis Algorithm for In Vitro Imaging of Triple-Negative Breast Cancer
Source: Nanomaterials (Basel). 2019 May 7;9(5):709. doi: 10.3390/nano9050709 (PMC6566234; doi:10.3390/nano9050709)
Supplement: Supplementary file 1 [file nanomaterials-09-00709-s001.pdf]

Supporting Information for

Targeted Gold Nanoparticle-Oligonucleotide Contrast Agents in Combination with a New Local Voxel-wise MRI Analysis Algorithm for *In Vitro* Imaging of Triple Negative Breast Cancer

Rajat Chauhan[1+], Nagwa ElBaz[4+], Robert S. Keynton[1*], Kurtis T. James[1], Danial A. Malik[1+], Mingming Zhu[3], Ayman ElBaz[1], Chin K. Ng[3], Paula J. Bates[2], Tariq Malik[2], Martin G. O' Toole[1*]

[1]Department of Bioengineering
[2]Department of Medicine and James Graham Brown Cancer Center
[3]Department of Radiology
[4]Department of Pharmacology and Toxicology
University of Louisville, Louisville, Kentucky USA

[+]Co-first authors; *Co-Corresponding authors

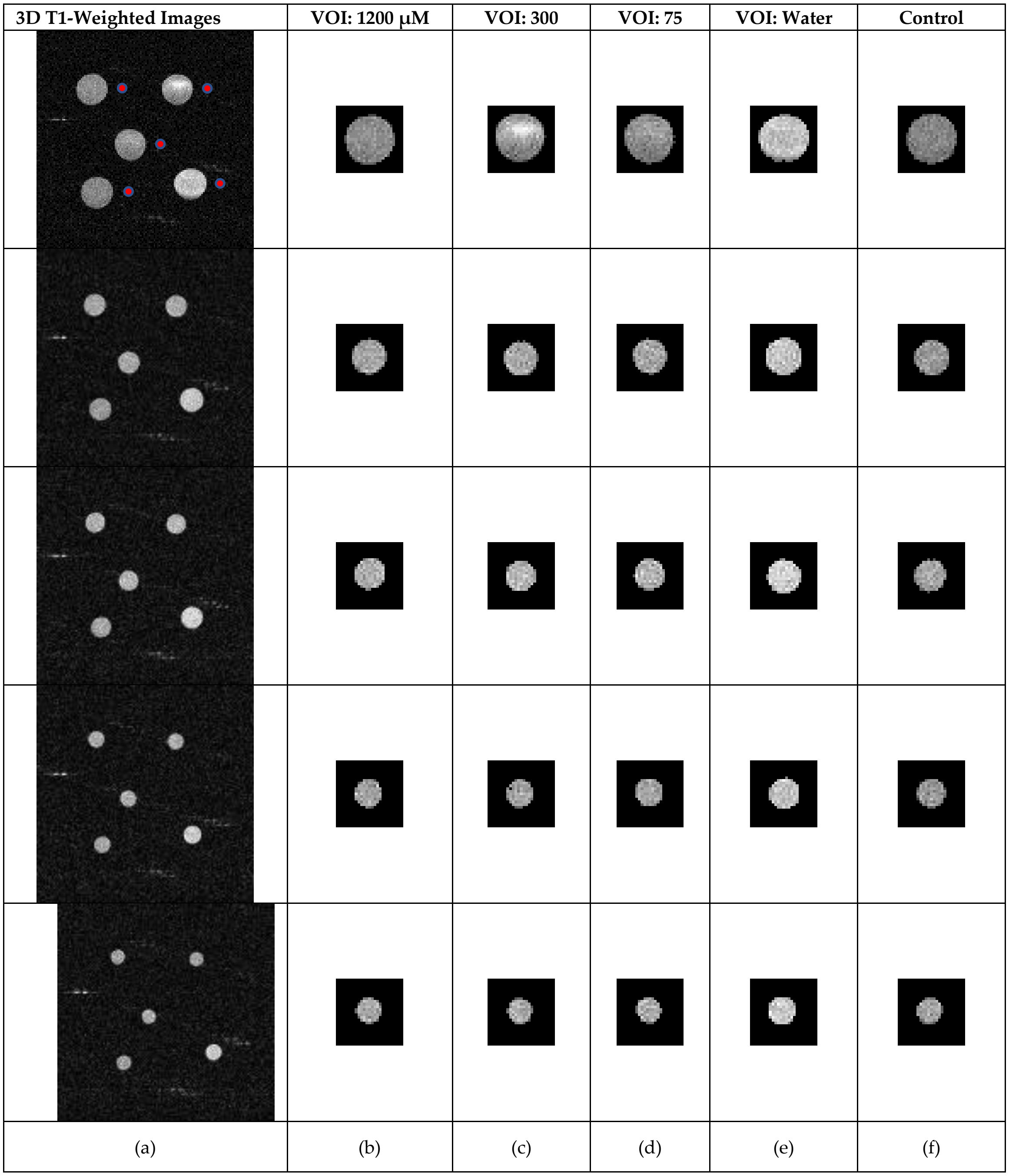

Figure S1. Illustration results of the automatic extraction of VOI.

Test (1200 μM)	Control (NTC)	Displaying the edges of test celles over the control before (red color) and after (green color) applying the registration steps.
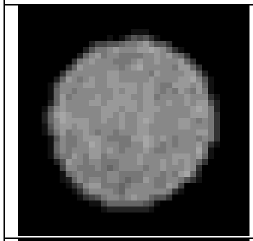	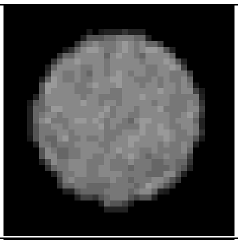	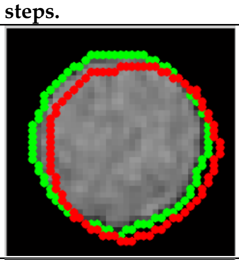
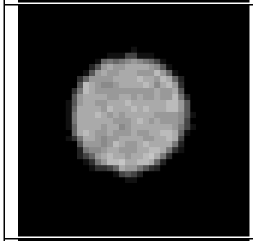	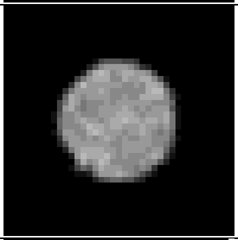	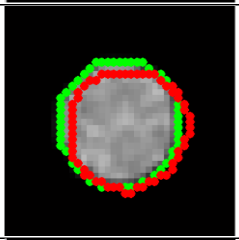
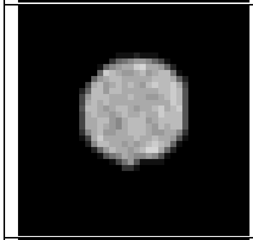	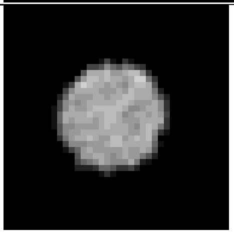	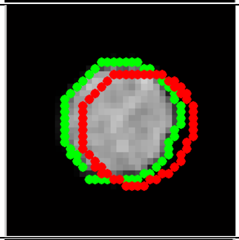
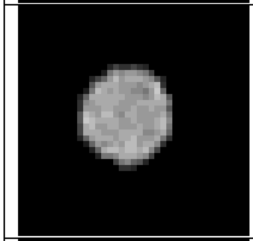	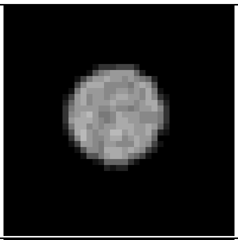	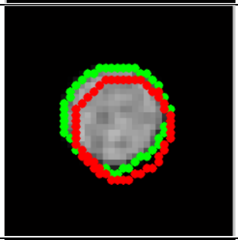
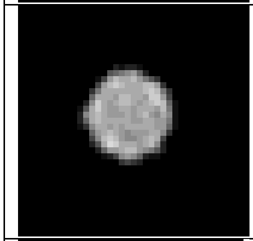	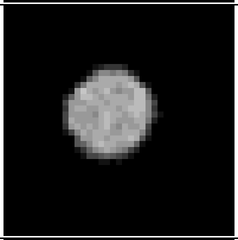	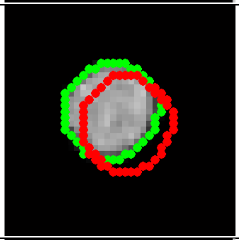
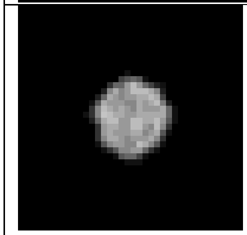	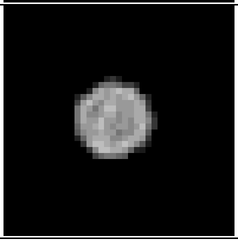	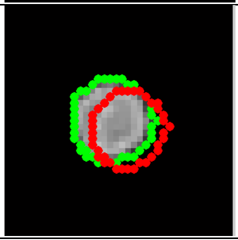

Figure S2. Illustration results of the non-rigid registration step.

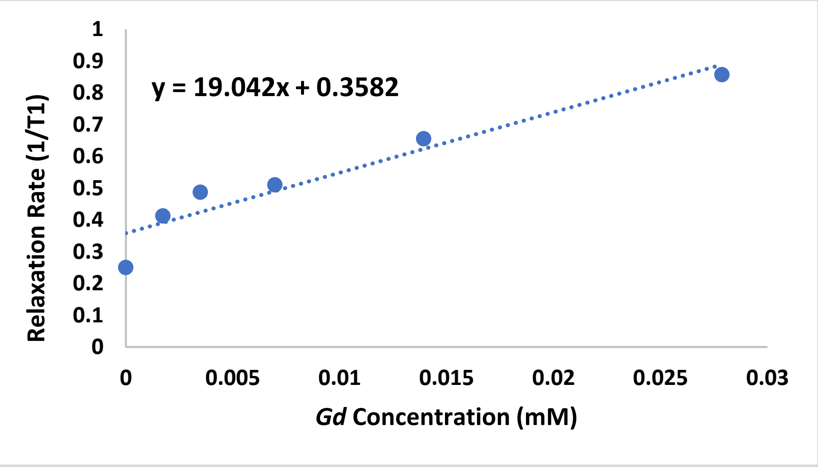

Figure S3. Relaxivity Curve for GNP-Gd(III) DO3A-SH-AS1411 in 0.7% agarose solution (9.4 T MRI scanner).

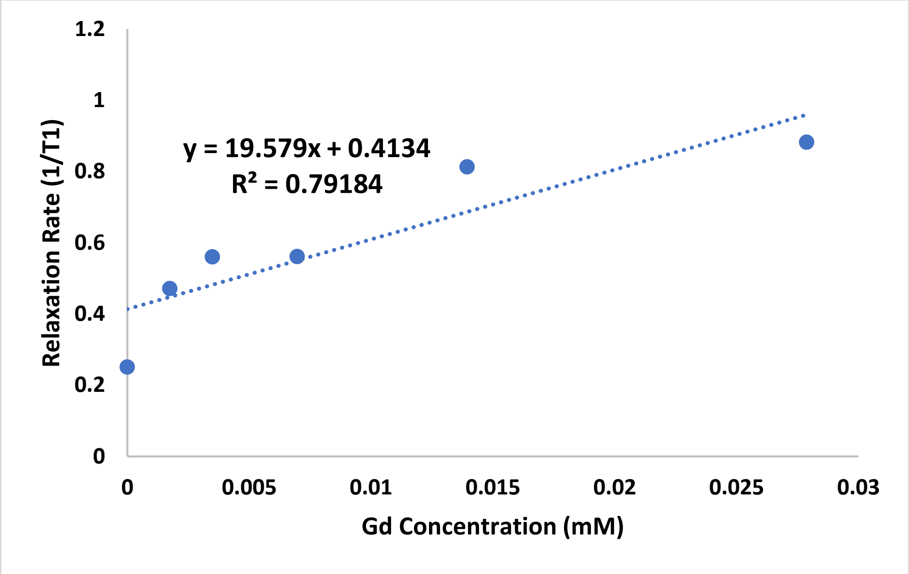

Figure S4. Relaxivity Curve for GNP-Gd(III) DO3A-SH-CRO in 0.7% agarose solution (9.4 T MRI scanner).

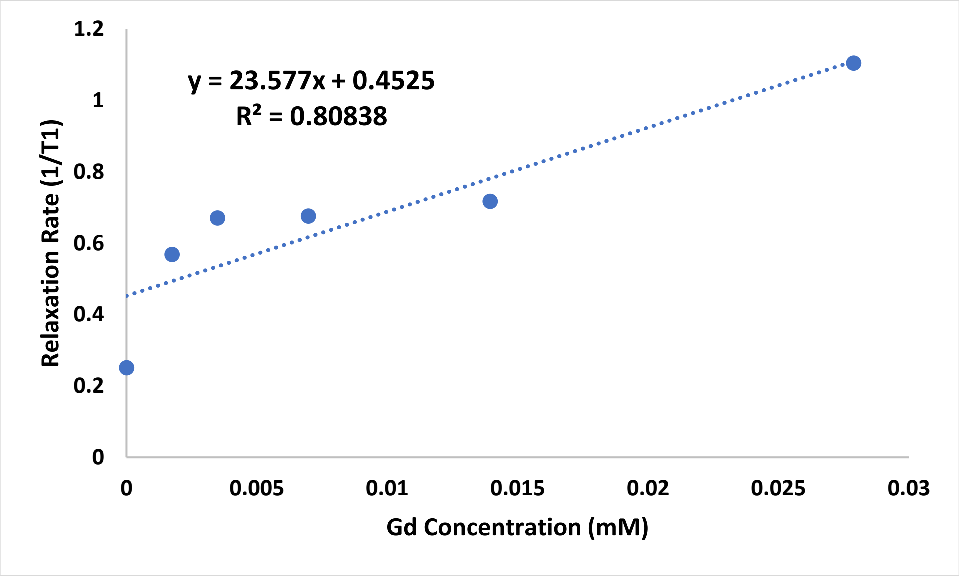

Figure S5. Relaxivity Curve for GNP-Gd(III) DO3A-SH-CRO in 0.7% agarose solution (9.4 T MRI scanner).

Table S1. Oligreen staining fluorescence values (FL) obtained for GNP Samples using Blue Module (*Excitation* 460 nm ; *Emission* 515-570 nm).

**Samples**	**Fluorescence (FLU)**
GNP *Gd(III) DO3A-SH* AS1411	3988.6
GNP *Gd(III) DO3A-SH*	37.5
GNP Gd(III) DO3A-SH AS1411 Filtrate	161.4
GNP *Gd(III) DO3A-SH* Filtrate	90.4
Oligreen 200X Solution	511.9
1 X PBS Buffer	74.6

Table S2. Gadolinium quantification per gold nanoparticle in GNP-Gd(IIII) DO3A-SH-Oligonucleotide.

**Sample**	**At% Au**	**At% Gd**	*# Gd(III) DO3A-SH*
GNP *Gd(III)-DO3A-SH*-AS1411	1.68	0.31	24.36
GNP-*Gd(III0DO3A-SH*-CRO	4.21	0.59	18.50
GNP-*Gd(III0DO3A-SH*-CTR	22.79	1.8	10.43

Table S3. Atomic and Weight percentages of elements analyzed in Energy Dispersive X-Ray Analysis of GNP Gd(III) DO3A-SH AS1411.

Element	*Wt %*	*At %*
C K	21.34	38.95
O K	16	21.93
NaK	27.57	26.29
AuM	15.06	1.68
ClK	14.95	9.24
K K	2.88	1.61
GdL	2.21	0.31

Table S4. Atomic and Weight percentages of elements analyzed in Energy Dispersive X-Ray Analysis of GNP-*Gd(III)DO3A-SH*-CRO.

Element	*Wt%*	*At%*
CK	27.13	52.39
OK	22.84	33.11
NaK	8.04	8.11
MgK	0.62	0.59
ClK	0.89	0.58
KK	0.67	0.4
GdL	4.02	0.59
AuL	35.78	4.21

Table S5: Atomic and Weight percentages of elements analyzed in Energy Dispersive X-Ray Analysis of GNP-Gd(III)DO3A-SH-CTR

Element	*Wt %*	*At %*
C K	7.85	40.77
SiK	15.6	34.64
AuM	71.99	22.79
GdL	4.55	1.8

Table S6. Gadolinium Quantification using xylenol orange Gd(III) protocol.

Gd content (# of atoms) per GNP-*Gd(III)DO3A-SH* -Oligonucleotide ; {Oligonucleotide = AS1411/CRO/CTR}		
AS1411	CRO	CTR
23.23 ± 0.93	19.21 ± 5.57	13.95 ± 0.73
